# A web-based intervention for patients with an implantable cardioverter defibrillator – A qualitative study of nurses’ experiences (Data from the ACQUIRE-ICD study)

**DOI:** 10.1016/j.pecinn.2022.100110

**Published:** 2022-11-28

**Authors:** Charlotte Helmark, Cecilie L. Egholm, Nina Rottmann, Søren J. Skovbakke, Christina M. Andersen, Jens B. Johansen, Jens C. Nielsen, Charlotte E. Larroudé, Sam Riahi, Carl J. Brandt, Susanne S. Pedersen

**Affiliations:** a Department of Cardiology, Zealand University Hospital, Roskilde, Denmark; b Department of Psychology, University of Southern Denmark, Odense, Denmark; c REHPA, The Danish Knowledge Centre for Rehabilitation and Palliative Care, Odense University Hospital, Nyborg, Denmark; d Department of Cardiology, Odense University Hospital, Odense, Denmark; e Department of Cardiology, Aarhus University Hospital, Aarhus N, Denmark; f Department of Cardiology, Copenhagen University Hospital Herlev-Gentofte, Hellerup, Denmark; g Department of Cardiology, Aalborg University Hospital, Aalborg, Denmark; h Steno Diabetes Center Odense, Odense, Denmark; i Research Unit of General Practice, University of Southern Denmark, Odense, Denmark; j Department of Clinical Research, University of Southern Denmark, Odense, Denmark; k Denmark and Department of Clinical Medicine, Aarhus University, Aarhus, Denmark; l Department of Clinical Medicine, Aalborg University, Aalborg, Denmark

**Keywords:** eHealth, CFIR, Implantable cardioverter defibrillator, Nursing role, Online communication

## Abstract

**Objective:**

The aim of this study was to explore cardiac nurses’ experiences with a comprehensive web-based intervention for patients with an implantable cardioverter defibrillator.

**Methods:**

We conducted an explorative qualitative study based on individual semi-structured interviews with 9 cardiac nurses from 5 Danish university hospitals.

**Results:**

We found one overall theme: “Between traditional nursing and modern eHealth”. This theme was derived from the following six categories: (1) comprehensive content in the intervention, (2) patient-related differences in engagement, (3) following the protocol is a balancing act, (4) online communication challenges patient contact, (5) professional collaboration varies, and (6) an intervention with potential. Cardiac nurses were positive towards the web-based intervention and believe it holds a large potential. However, they felt challenged by not having in-person and face-to-face contact with patients, which they found valuable for assessing patients’ wellbeing and psychological distress.

**Conclusion:**

Specific training in eHealth communication seems necessary as web-based care entails a shift in the nursing role and requires a different way of communication.

*Innovation*

Focusing on the user experience in web-based care from the perspective of cardiac nurses is innovative, and by applying implementation science this leads to new knowledge to consider when developing and implementing web-based care.

## Highlights

• Cardiac nurses are positive towards web-based treatment.
• Online communication entails a shift in the nursing role and requires training.
• Web-based treatment can challenge assessment of patients’ psychological wellbeing.

## 1 Background

An implantable cardioverter defibrillator (ICD) is the treatment of choice for primary and secondary prevention of sudden cardiac death [1]. The ICD treatment is for patients at high risk of a cardiac arrest due to ventricular arrhythmia or severe heart failure [1]. The ICD is an advanced implantable device, usually placed under the skin in the left shoulder region, with leads connecting to the vasculature of the heart. The ICD monitors the heart rhythm and will induce shock therapy in case of a life-threatening arrhythmia. Although patients generally adapt well to life with an ICD, 20% are challenged psychologically and develop anxiety, depression, or posttraumatic stress. Fear of shocks and actual shocks may induce avoidance behaviours, leading to a sedentary lifestyle in some patients [2]. Psychological distress is associated with poor health-related quality of life (HRQoL) [3,4] and increased risk of mortality [5,6]. Hence, it is paramount to identify and treat these patients for their psychological distress to improve HRQoL and associated health outcomes.

Patients with an ICD are primarily monitored remotely [3], reducing in-person contacts with cardiac nurses [7]. With less interaction there is a greater risk that psychological distress will go unnoticed and untreated [8].

New technology allows for web-based interventions enabling patients to engage in psychological and supportive interventions regardless of time and place. Studies have shown that web-based solutions can be beneficial for cardiac patients [9,10]. A comprehensive web-based intervention aiming at supporting patients in their transition to life with an ICD is currently being evaluated in a randomised controlled trial (RCT), with the intervention being delivered by cardiac nurses [3].

Alongside with patients, cardiac nurses are important stakeholders in eHealth - such as web-based interventions - as implementation of new technology may be associated with provider-level barriers [11]. While cardiac patients’ experiences with web-based interventions have been reported previously [[12], [13], [14]], knowledge of how cardiac nurses experience web-based care is sparse [15]. One study reported that an e-learning platform for motivational interviewing was acceptable to cardiac nurses [16]. The results were questionnaire-based, and the authors suggest further research to improve the understanding of health care professionals’ engagement with web-based treatment. Another study evaluated a cardiac nurse-led web-based intervention targeting medication adherence but did not include nurses’ perspectives [17]. A recent study explored perceived barriers and facilitators for eHealth solutions regarding lifestyle change among health care professionals in cardiac care [18]. They recognized potential advantages of eHealth, but also voiced concerns about usability due to the older age of cardiac patients and the belief that patients prefer in-person communication [18].

Implementation science states that adaption of new technologies, such as web-based interventions, into clinical practice is complex, and may be associated with a range of barriers that influence the implementation [19]. Applying a theoretical approach based on implementation science to studies gives the opportunity to better understand factors that influence implementation [20]. Implementation science claims that individual stakeholders can play a crucial role in the process of transitioning new interventions into clinical practice, since they hold attitudes and beliefs about the intervention, make choices and can influence others with both predictable and unpredictable consequences [19]. For instance, nurses might refrain from including older patients if they believe that the intervention is not appropriate for this subgroup as previously reported [18]. Therefore, it is highly relevant to explore the dynamic interplay between the individual stakeholders in terms of cardiac nurses and the current web-based intervention to better understand potential barriers for future implementation. Thus, the aim of this study was to explore cardiac nurses’ experiences with a comprehensive web-based intervention for patients with an ICD.

## 2 Methods

This study was conducted using an explorative qualitative design based on semi-structured interviews and qualitative content analysis [21]. Qualitative research is suitable for exploring complex phenomena as encountered by individuals and thereby contribute with meanings in a broad sense [22]. We chose an explorative approach, as only limited literature was identified that was relevant for the study aim, meaning we had to keep an open mind in our data-driven analysis. The study is part of a larger RCT - the ACQUIRE-ICD [3] - and focuses on cardiac nurses’ experiences in the intervention arm of the study. Reporting of the study is guided by the Consolidated Criteria for Reporting Qualitative Research (COREQ) [22].

### 2.1 Setting

This nation-wide study took place across five Danish university hospitals. ACQUIRE-ICD – A personalized and interactive web-based health care innovation to AdvanCe the QualIty of life and caRE of patients with an ICD [3] is a comprehensive intervention developed to facilitate the transition to life with an ICD. The 12-month multi-component intervention includes systematic dialogue with cardiac nurses; educational material; goal setting for behavioural change; monitoring and treatment of anxiety and depression; and access to an online patient forum (Table 1). The ACQUIRE-ICD intervention was delivered by cardiac nurses [3], who received training before study initiation, mainly in the manual and practicalities of delivering the intervention.

**Table 1:** Overview of the components of the 12 months ACQUIRE-ICD intervention.

Written communication with cardiac nurses on the platform	Weekly 1-3 months post ICD implant (nurse-initiated) Monthly 4-12 months post ICD implant (nurse-initiated) Extra dialogue possible, both written and by telephone (nurse- or patient initiated)
Information provision and patient education	Toolbox of material distributed to patients at regular intervals (see above). For instance, material on ICD-related topics, anxiety, depression, behavioural change, sleep, relaxation training, quizzes, podcasts with cardiac patients and health professionals.
Monitoring of anxiety, depression, and self-rated health	Monthly (tools were GAD-7 for anxiety, PHQ-9 for depression and EQ-VAS for self-rated health)
Referral for psychological treatment in case of elevated scores for anxiety and/or depression	Cognitive behavioural therapy delivered by psychologists
Goal-setting for behavioural change	Minimum one goal was required upon entrance to the platform, with no upper limit of goals (e.g., improvement in activity level, smoking cessation, or diet change). This action was a requirement from the chosen platform.
Online patient forum.	Patient-to-patient communication - no moderation by health professionals (the data manager could access the forum and redirect in case of negative conversation).

Abbreviations: *ICD* Implantable cardioverter defibrillator; *GAD* Generalized Anxiety Disorder scale; *PHQ* Patient Health Questionnaire; *EQ-VAS* EuroQol visual analogue scale.

The intervention was delivered weekly for three months and monthly for the following nine months. It included sending personalized material to patients and contacting non-respondent patients to enhance adherence and patient motivation. The nurses could via the intervention platform assess whether patients had been active or not. In the asynchronous chat, patients could ask questions or request specific material, and nurses could send encouraging messages. Patients also received feedback on the questionnaires from the nurses, with the possibility to reflect on this. The nurses could contact patients by phone in case of non-adherence or concern for patients’ wellbeing.

Concurrently with the intervention tasks, the nurses had various employment in their hospitals as e.g., study nurses or ICD nurses.

### 2.2 Theoretical framework

The study was inspired by the Consolidated Framework for Implementation Research (CFIR) [19]. The CFIR is a meta-theoretical framework with a comprehensive range of constructs related to five domains that may influence implementation: the implementation process, the individuals, the intervention, inner setting, and outer setting (Table 2). We found CFIR suitable for this study as it contains a domain about the individuals involved in delivering the intervention, including perceived needs and resources of patients. The CFIR is based on theories of organizational change beginning with individual change. This means, that the individuals are a key feature in implementation, and the framework is therefore relevant in the context of this study regarding individuals delivering a new type of intervention [20].

**Table 2:** Brief overview of the Consolidated Framework for Implementation Research.

Domain	Description
Intervention characteristics	Relates to the components of the intervention being implemented in terms of evidence, advantages, adaptability, and the quality of design. As an intervention is often complex, core and peripheral components can be assessed individually.
Outer setting	This domain covers the economic, structural, and societal context around the organization, including the patient’s perspectives.
Inner setting	The cultural and political climate inside the organization where the implementation happens. This includes constructs such as available resources, incentives, communication, and networking.
Characteristics of individuals	Encompasses the individuals involved with the implementation and relates to the fact that individuals have power to influence others as they carry mindsets, interests, and norms.
Process	Relates to the active change processes that aim at achieving implementation and often consists of a range of sub-processes that might be planned or occur spontaneously.

Based on Damschroeder et al 2009.

Noticeably, the five domains interrelate and can therefore overlap.

CFIR inspired the interview guide, while the analysis took an inductive approach. Relevant CFIR domains were applied in the discussion as theoretical inspiration with respect to our results.

### 2.3 Participants

We used purposive sampling to ensure relevant experience with the intervention. The nurses (n=9) were identified through the ACQUIRE-ICD project team and invited by email. They received an information letter describing the purpose of the study, alongside with the informed consent. As all invited nurses accepted, we had a complete sample of possible informants.

All participants were cardiac nurses (one male, eight females) with routine in care for patients with an ICD. They had from 2-23 years of experience with ICD patients with an average of 11 years. Most were involved in all aspects of the intervention, while two were involved only in either recruiting patients or supporting patients online.

### 2.4 Interview guide

The interview guide was based on empirical and theoretical knowledge, including the CFIR framework [19]. The focus of the interview guide was experiences with delivering the intervention; the content of the intervention; and collaboration. We aimed to keep the questions explorative to let the nurses talk about their experiences without guiding them in any predefined direction. Follow-up questions were prepared in case the informants needed guidance or exemplification. The interview guide (Appendix A) was pilot tested on the target group and minor adjustments added.

### 2.5 Data generation

We conducted individual semi-structured interviews, as these are appropriate for generating knowledge on personal experiences of topics [22]. We chose individual interviews to gain as many detailed and broad experiences as possible, without the participants influencing each other’s reflections [19]. Due to COVID-19 restrictions in-person interviews were not feasible. Instead, the nurse could choose between video- or telephone interview which are considered trustworthy and valid alternatives [23]. Six chose telephone interviews, while two chose video interviews. One interview was conducted in-person upon request of the interviewee. The interviews took place in May/June 2021 and lasted 17-50 minutes. All interviews were audio-recorded.

The interviews were conducted by the first author (CH). She is an experienced cardiac nurse and PhD student with routine in interviewing who could establish a connection with the informants due to shared backgrounds as cardiac nurses. The establishment of a good connection aimed to achieve sufficient information power [24].

### 2.6 Analysis

**Fig. 1:**
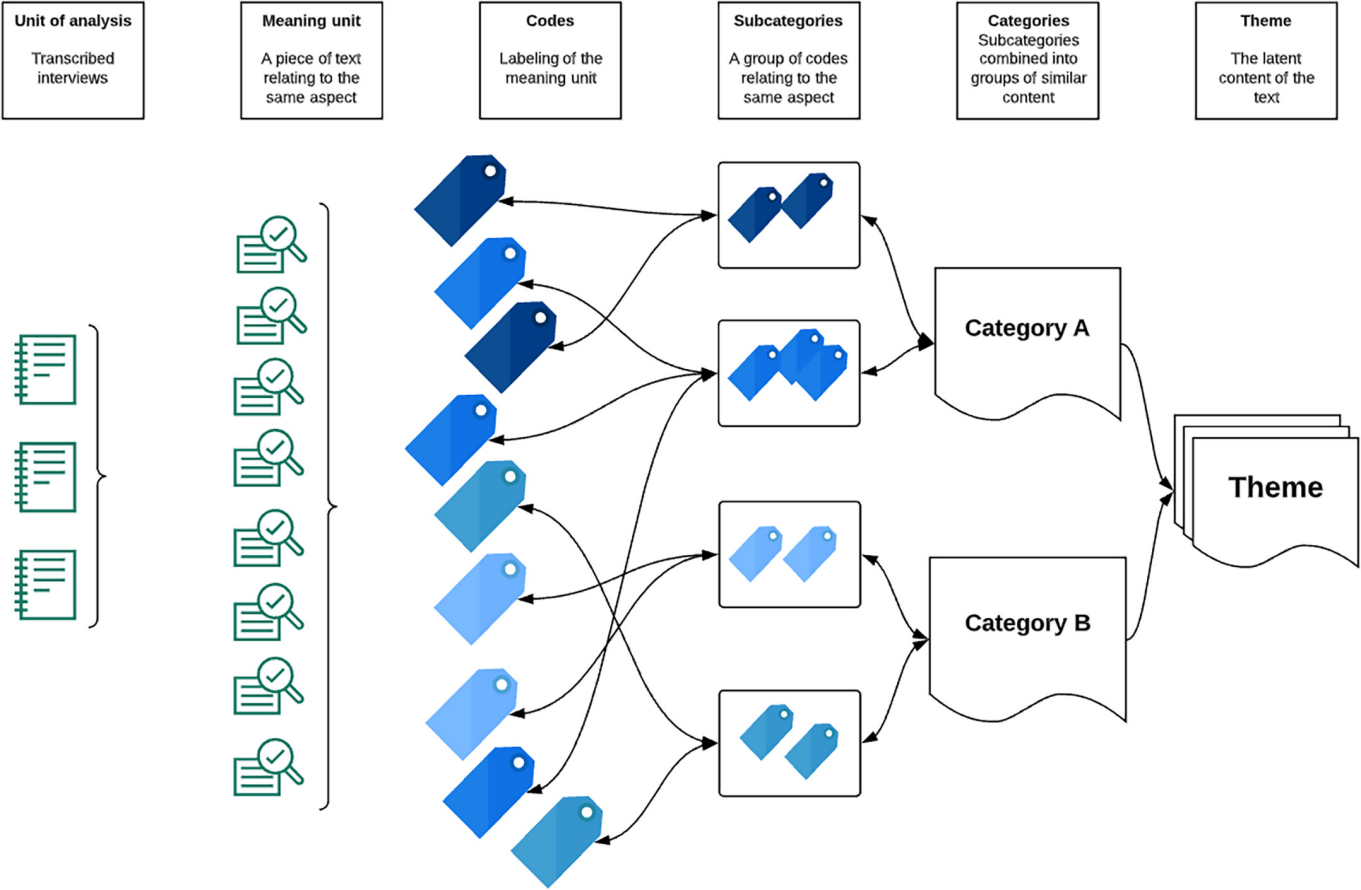
Illustration of qualitative content analysis, inspired by Graneheim and Lundmann.

Data were analysed using qualitative content analysis with an inductive approach [21]. The interviews were transcribed verbatim by the first author (CH). By using researcher triangulation, we strived to illuminate both mutual confirmation and different perspectives of codes. Two authors (CH and CL) repeatedly read the transcripts and coded the same three transcripts independently. Thereafter, the codes were discussed in-depth until consensus of coding was reached. A manual for coding was developed, the remaining transcripts were coded according to the agreed manual, thus adding breadth and reliability to the further analysis. To increase trustworthiness two additional authors were included in the next analytical steps (NR and SSP). The codes were reflected upon several times and grouped into subcategories relating to the same area. Hereafter, the subcategories were combined into categories, which describe a similar content in relation to the study aim. The process of creating subcategories and categories was dynamic, reflecting back and forth several times. When the categories were finalized, we discussed what unified these and derived an overarching theme (Fig. 1). Examples can be seen in Appendix B. Data management was conducted with the use of NVivo version 12. The findings were analysed and reported with thick descriptions to gain transferability. We strived to maintain an inductive approach throughout the analysis.

### 2.7 Ethics

The study was approved by the Danish Data Protection Agency at the University of Southern Denmark (11.380). Ethics approval was obtained from the Research Ethics Committee at the University of Southern Denmark (21/27575). The study complies with the Helsinki Declaration with all participants providing written informed consent. To protect confidentiality of the nurses, age and hospital names are not reported.

## 3 Results

### 3.1 Theme

**Fig. 2:**
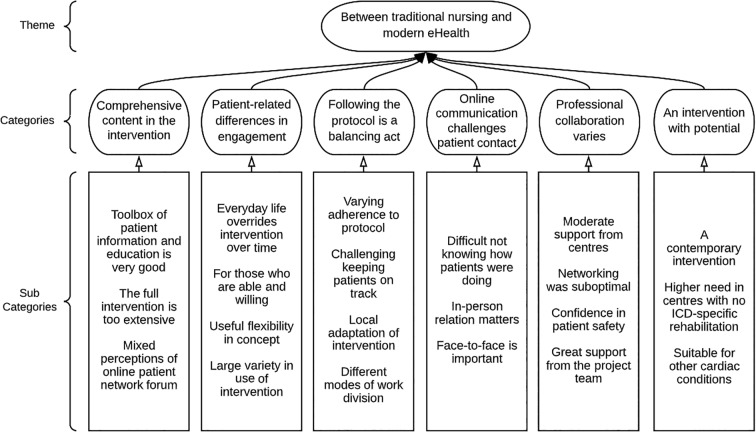
Theme, categories, and subcategories.

We identified an overarching theme “Between traditional nursing and modern eHealth”. This theme unifies the six categories, each covering various subcategories (Fig. 2). The theme covered the latent content [21] of the data-driven analysis and suggested that the nurses were split between traditional nursing values and the promises of new technology. On one hand they were positive towards the web-based intervention and believed it holds great potential. On the other hand, they were challenged by limited face-to-face contact, which they found valuable for assessing patients’ psychological wellbeing. This indicates an unsolved disharmony that will be described in the following.

### 3.2 Comprehensive content in the intervention

All nurses were positive towards the toolbox of information- and educational material, which they found well equipped and of high quality. This allowed for choosing material according to the needs and preferences of patients.

However, the nurses found the full intervention too comprehensive, as they experienced that some patients were overwhelmed with the wide range of tasks. Most nurses did not perceive goal-setting to be relevant, pointing at adjusting to life with an ICD to be patients’ primary target.

➢ *Informant 3: It is too many things to handle. They should not have to both focus on weight loss and having suffered a cardiac arrest, they should focus on one thing at the time. And we prioritize dealing with the cardiac arrest first, then weight loss can follow later. You can’t cope with it all at the same time, it is too much for the patient.*

It was difficult for some nurses to relate to the online patient forum as part of the intervention because they were not in control of the communication. They worried about misinformation and tone of communication yet feeling responsible, as it was part of the intervention.

➢ *Informant 8: And of course, it is important that you don’t make an online networking forum where there is free room for everything in case there are matters that are profound false and could scare somebody.*

Other nurses were positive about connecting a group of patients and letting them communicate without interference.

### 3.3 Patient-related differences in engagement

The nurses experienced a large variety in patients’ extent of use of the intervention, yet all nurses experienced that patients generally lost interest in the 12-month intervention concurrently with returning to daily life and adapting to life with an ICD. Over time, patients more often forgot to respond to questionnaires and used the materials less. The nurses found this understandable, as everyday life slowly overcame thoughts and emotions about illness.

➢ *Informant 4: But they were living their lives and it is really nice that they have their everyday life. Everyday life beats looking at the computer.*

The nurses experienced that the intervention fits best to patients with some level of resources as a surplus of energy is required to engage. Especially, some nurses were concerned about older patients and those with limited IT literacy as they believed these subgroups would have difficulties participating. Otherwise, the nurses believed that the intervention was suitable for most patients. They pointed out that patients who had suffered a cardiac arrest were easier to include and seemed to have a higher need for the intervention compared to patients who had received the ICD prophylactically.

➢ *Informant 1: But there were also those with cardiac arrest, and I believe there is a huge difference in needs. This middle-aged man who had been hugely active, had a cardiac arrest and was resuscitated. He definitely had different needs than those who receive it (the ICD) because they POTENTIALLY were at higher risk.*

The nurses were positive towards the flexibility of the intervention because it allowed patients to engage on the platform whenever patients preferred. They found the web-based format an advantage for patients travelling long distances, as these patients might otherwise decline cardiac rehabilitation interventions. They also experienced that the intervention gave patients good opportunities to engage with relatives, as they could share educational material, for instance by watching a video together.

### 3.4 Following the protocol is a balancing act

The nurses reported having followed the protocol regarding delivery of material to patients, but for most centres restricted resources led to irregular communication periodically as well as local adaptation of procedures. Competing clinical tasks further necessitated adjustments in intervention delivery.

It was difficult for the nurses to assess how much they should chase responses from patients to keep them adherent to the intervention. The nurses worried about appearing overzealous if they kept contacting patients, feeling the protocol made them push too hard.

➢ *Informant 7: I felt they became annoyed at you when you called, right? (laughs). Like you are overzealous, you could feel that sometimes. Of course, they should not have that experience.*

The nurses adjusted and improved the procedures locally, so it became more meaningful for them.

➢ *Informant 8: Of course, there was mandatory information, but there were options I could ignore, for instance smoking cessation when the patient didn’t smoke. Things like that. I thought it was cool that I could influence it myself (…). And it means something, that you can adjust it along the way, because otherwise I’m sure we would have lost some of the patients.*

As the tasks were conducted differently at the five participating centres, the nurses believed that this could impact local outcomes.

### 3.5 Online communication challenges patient contact

Most nurses found it difficult that they lost touch with how the patients were doing. For instance, when a patient did not reply in a chat, they were in doubt if the patient was unwell or just forgetful. The nurses perceived it as sending messages into cyberspace without knowing if it reached the patient. Few nurses felt it was the patients’ own responsibility to act if they needed help.

➢ *Informant 4: You could have this feeling that you send stuff out in the air and that’s it (laughs). You see some have bad scores on anxiety and depression, and you try to write to them, and they do not respond.*

Some nurses felt that they lacked competences in online written communication with patients. They reflected on the balance between using everyday language supplemented with emojis to better connect with patients versus using a language equal to documentation in electronic health records.

➢ *Informant 8: It has actually been a bit difficult because you have not been well trained to chat. Where you can say, now they have this in writing, and they can take it out and complain about it. It is a different mind-set. Of course, you should stand by what you say, but you have a different mindset when it is there, black on white. I believe you should really be careful what you write in the chat. Therefore, this has ALSO been difficult; I have used a lot of time reflecting on this.*

Most nurses found it important to have a personal relation with the patients. They believed that it enhances communication and adherence when patients have “a face” to relate to.

The intervention brought about an insight that in-person communication is important because of the nuances in non-verbal communication. Particularly, the nurses perceived online communication to be a barrier for venting emotional aspects, as they found that patients had difficulties expressing themselves in writing about this.

➢ *Informant 5: I think sometimes in this study, which is much about psychological dimensions, you can miss out on things because you can´t see the patients or their body language, mimic and so on compared to when you have them face-to face.*

They suggested face-to-face could be conducted on video, keeping the advantages of the online intervention.

### 3.6 Professional collaboration varies

The nurses experienced moderate support from their local hospital. They experienced concurrent studies initiated by industry or medical doctors had higher priority, possibly due to financial aspects.

The nurses did not experience good networking opportunities to share knowledge and discuss improvements of procedures. Some nurses missed this while others did not. Most nurses were aware of project meetings scheduled by the project team, but most were not able to attend these due to competing clinical tasks.

Regarding patient-safety, the nurses experienced quick response from the psychologists, in case of referral due to elevated screening scores for anxiety and/or depression. However, some nurses lacked interdisciplinary communication with the psychologists to optimize collaboration around the individual patient.

➢ *Informant 8: I have never talked to a psychologist about this, and I´m not sure what they are doing. However, I can see they put something on the platform and run some sort of side-communication that seems a bit drastic with a lot of homework to the patients.*

All nurses experienced that the project manager has been highly capable and available for enquiries related to technical issues and procedures.

### 3.7 An intervention with potential

The nurses found the intervention innovative, and were confident that online intervention is the future, particularly since the COVID-19 pandemic emerged towards the end of the trial period. They experienced how new technical solutions in the healthcare system were made possible rapidly, and that patients were positive towards this transition and expected digital solutions:

➢ *Informant 5: Regarding participant information there were actually quite a few – also from the much older generation – who say, “But I have an email, can’t you just email me?” (Laughs). I sometimes feel a bit outdated, for that idea hadn’t crossed my mind”.*

Dependent on concurrent local ICD specific offers to the patients, the nurses had differentiated attitudes to the relevance of the intervention. For centres who already had a local model for ICD rehabilitation, the nurses did not perceive it as relevant compared to nurses from centres with no ICD specific offers.

All nurses found that with diagnosis-specific adjustments, the intervention could be used for other cardiac conditions such as atrial fibrillation or heart failure.

## 4 Discussion and conclusion

### 4.1 Discussion

In this qualitative study of nurses’ experiences with a web-based intervention, six categories were identified, and the essential aspects of the categories were unified in the overarching theme “Between traditional nursing and modern eHealth”. The theme illuminated the cardiac nurses’ reflections of having positive attitudes towards web-based treatment and believing in this being a future mode of care delivery while at the same time being challenged by lacking face-to-face contact for assessment of patients’ psychological wellbeing and worried about consequences for less resourceful patients.

#### 4.1.1 Lack of personal contact

We found that lack of in-person or face-to-face contact were the biggest challenges in online care delivery for the nurses. Relying on written online communication made it difficult for nurses to assess patients’ well-being and to tackle non-respondent patients. This mirrors previous reports of challenges in eHealth, contrasting face-to-face communication with immediate response [25]. Especially, we found that nurses were challenged in the assessment of patients’ psychological wellbeing, as they could not interpret verbal and non-verbal communication, which they are trained to do traditionally. In CFIR, “Characteristics of Individuals” reflects that skill in the use of an intervention relies on adequate how-to knowledge and may influence implementation [19]. Likewise, previous studies emphasise the importance for healthcare providers to have the necessary skills for safe eHealth delivery [26,27]. In our study, the nurses had no prior experiences with replacement of in-person communication and became insecure, since they felt responsible for the patients’ care, indicating suboptimal training. How-to knowledge was also requested from the nurses on how to balance text in the chats, so it became personal for the patients but still appeared professional. However, looking at the context of our study, the ACQUIRE-ICD study began in 2017 [3], which was years before the COVID-19 pandemic, and development of eHealth in the cardiac field has since accelerated rapidly along with new insights and possibilities like video consultations [26,28].

Since the nurses in the current study expressed lacking personal relation and face- to-face communication to ensure a patient-centred approach, hybrid solutions may be a solution. This, since hybrid solutions are add-ons to usual care instead of replacements, giving better opportunities to build relationships [18,25]. A position paper from the European Society of Cardiology states that shifting roles and responsibilities are barriers that need to be addressed when implementing eHealth [29]. The CFIR domain “Intervention Characteristics” describes that the perception of relative advantage of an intervention influences implementation [19]. Hence, if nurses experience web-based solutions a disadvantage towards patient-centred care, this might be a substantial barrier for successful implementation. To enhance nurses’ skills and understanding of eHealth delivery, it seems paramount to develop a training program that will make them feel competent to take on this task [28,29].

The possibility of integrating a face-to-face option in the ACQUIRE-ICD intervention may be the missing link that would improve the relationship between nurses and patients and be perceived an aide for the cardiac nurses. Thus, while the chosen treatment platform was considered the best available option at initiation of the study, rapid development of web-based solutions speaks in favour of switching to a more suitable platform in case of future implementation of the intervention [30].

#### 4.1.2 Appropriateness of intervention

We found that older age and lack of resources were perceived barriers among the nurses regarding patient participation and adherence. Such barriers have been reported previously [18] and could potentially deprive these subgroups of effective eHealth interventions in case healthcare professionals are reluctant to include them. However, a recent study found that older age was a predictor for optimal motivation and adherence to an app-based intervention for heart rate and rhythm monitoring [31]. Despite that the mentioned study [31] might be a less complex intervention than ACQUIRE-ICD, it still emphasizes that we should not underestimate digital health competencies among older patients. Developers of eHealth interventions should also address age- and literacy-related barriers through better introduction and user-friendly platforms to enhance uptake [29]. The CFIR domain “Characteristics of Individuals” claims that knowledge and beliefs among those involved in delivering an intervention are important factors for implementation [19]. The nurses in our study found the intervention to be more appropriate for patients who had suffered a cardiac arrest than patients without a history of cardiac arrest, indicating that they were aware of differentiation in needs but were not capable of translating this into tackling for instance patients with older age.

All nurses worried about the appropriateness of the intervention for older patients, due to potentially low eHealth literacy. eHealth literacy is often defined as *“the ability to seek, find, understand, and appraise health information from electronic sources and apply the knowledge gained to addressing or solving a health problem”* [32]. As eHealth interventions are increasing, the understanding of eHealth literacy is paramount, and research in eHealth literacy in the cardiac field is increasing [33]. In one study of a cardiovascular risk population, there was no association between sociodemographic factors and eHealth literacy, but spending >1 hour daily on the internet was associated with high level of eHealth literacy [33]. Another study of eHealth literacy among patients undergoing percutaneous coronary interventions found only a weak correlation with age [34]. A third study on cardiac patients’ experience with cardiac telerehabilitation did not find an association between age and eHealth literacy, but that the web-based intervention improved patients’ eHealth literacy [9]. This indicates again that health professionals should not refrain from including patients in eHealth solutions due to older age. Another solution could be to tailor the web-based interventions to meet cardiac patients’ individual needs and preferences by for instance developing user-friendly interfaces or differentiate the content to various age groups. From the CFIR domain “Characteristics of Individuals” we know that individuals’ knowledge and beliefs are important factors for implementation, and in addition that opinions obtained from peers are convincing [19]. So, training in online treatment could include having a trustworthy peer sharing knowledge of older patients’ eHealth. Otherwise, it may be the cardiac nurses themselves that act as barriers for successful implementation.

#### 4.1.3 eHealth innovations

The nurses in our study found the intervention - and eHealth in general - innovative, and that it has great potential, mirroring previous findings in the cardiac field [[28], [29], [30]]. In the CFIR domain “Intervention Characteristics” it is emphasized that if the benefits of the intervention are clearly visible to stakeholders, it will influence implementation positively [19]. The nurses experienced that many patients were ready for transition to eHealth, especially towards the end of the study where the COVID-19 emerged, where patients had tried for instance video consultations with their general practitioner. Considering pandemics like COVID-19, eHealth has a large advantage compared to on-site care, optimizing chances of successful implementation according to CFIR [19].

The nurses were positive towards the flexibility of asynchronous care, as it gave autonomy to patients to choose when to engage. This is previously reported [18], also from the patient perspective [14], and therefore seems an important facilitator at both patient- and provider level [15].

We found divergent perceptions of the online patient forum. This mirrors previous studies suggesting that a moderated online forum can be supportive for cardiac patients [35], but barriers such as unwillingness to share personal experiences online are also reported [14,35]. This indicates that evaluations of online patient forums are still needed to find evidence-based models matching cardiac patients’ needs and preferences.

Involving patients in the development of new interventions through Patient and Public Involvement strategies has become mainstream, as patients are the obvious and most important end-users [36,37], and this was also done in ACQUIRE-ICD [38]. Nevertheless, involvement of other key stakeholders in the development of interventions – such as the cardiac nurses – may enhance implementation. The CFIR domain “Characteristics of Individuals” claims that all involved individuals carry professional and individual mind-sets and through a dynamic interplay they will seek to find a meaning with the intervention and try to improve or adjust it [19]. Thus, also involving the nurses delivering the intervention in the development phase may be important to optimise fidelity to the intervention during initial testing as well as later successful real-life implementation. Based on the findings of our study, we recommend offering e.g., regular webinars or workshops to involved nurses to counter local deviations from the intended intervention and discuss cases and experienced challenges.

### 4.2 Strengths and limitations

A major strength of this study is that all invited nurses agreed to participate, representing experiences across all participating centres. It could be considered a limitation that only nine interviews were conducted. However, during the interviews, the nurses provided broad and thorough descriptions of their experiences. The nurses were dedicated and well-articulated which led to clear communication and achievement of sufficient information power [24].

The interviewer was a cardiac nurse which could be considered both a strength and a limitation, since the understanding of cardiac nursing was good while other perspectives might have been overlooked. However, to counterbalance possible bias, co-authors with other backgrounds (expert in implementation science (CL) and psychologists with expertise in qualitative research (NR/SSP)) were deeply involved with the interview guide and analysis. A limitation of the study is that we explored nurses’ experiences with an experimental intervention, potentially reducing transferability to clinical practice as experiences there might have led to other findings due to different stakeholders and resources [15]. Still, we believe our findings to be important when planning future eHealth interventions involving cardiac nurses.

### 4.3 Conclusion

Cardiac nurses were positive towards the concept of web-based interventions, but also lacked face-to-face contact with patients, especially when assessing psychological distress. Ensuring face-to-face contact by either personal contacts or video might enhance the value of web-based interventions from cardiac nurses’ perspective. Specific training in eHealth communication seems necessary as web-based care entails a shift in the nursing role and requires a different way of communication.

### 4.4 Innovation

Web-based care is an innovative mode of treatment, and it is likely to increase in the future. In addition, focusing on the user experience in web-based care from cardiac nurses’ perspective is also innovative. By further applying implementation science to the study, this leads to new knowledge regarding development and implementing of future web-based care.

## Author contributions

CH, CLE, NR and SSP designed the study, performed the analysis, and drafted the manuscript. SJS, CMA, JBJ, JCN, CL, SR, and CJB critically revised the manuscript and provided feedback. All authors approved the final manuscript before submission.

## Funding

The ACQUIRE-ICD study was supported with a seeding grant from Patient@home, University of Southern Denmark and grants from the Lundbeck Foundation and Trygfonden.

Statement

I confirm all patient/personal identifiers have been removed or disguised so the patient/person(s)

described are not identifiable and cannot be identified through the details of the story.

## Declaration of Competing Interest

Susanne S Pedersen reports financial support was provided by Lundbeck Foundation. Susanne S Pedersen reports financial support was provided by TrygFonden.

## Appendix A Supplementary data

Interview guide Image 1

Coding process Image 2
