# Supplementary material for: A web-based intervention for patients with an implantable cardioverter defibrillator – A qualitative study of nurses’ experiences (Data from the ACQUIRE-ICD study)
Source: PEC Innov. 2022 Nov 28;2:100110. doi: 10.1016/j.pecinn.2022.100110 (PMC10194258; doi:10.1016/j.pecinn.2022.100110)
Supplement: Supplementary file 1 — Interview guide [file mmc1.docx]

INTERVIEW GUIDE


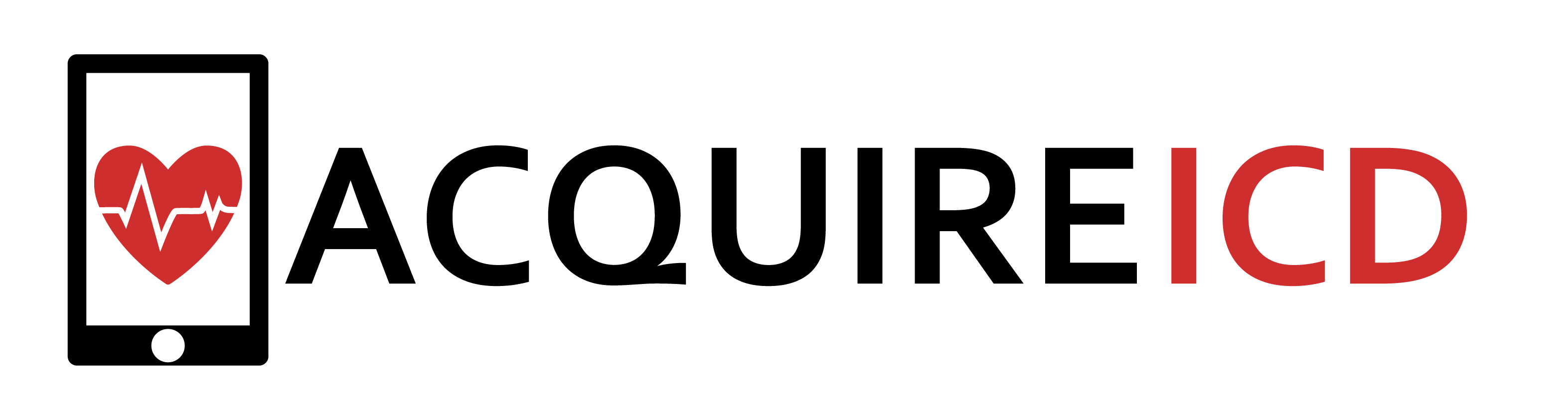


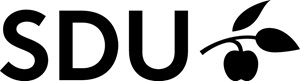


**Interview of nurses on their experiences with the ACQUIRE - ICD intervention**

| **Subject** | **Interview questions** | **CFIR domain/notes** |
| --- | --- | --- |
| **Introduction** |  |  |
| Presentation of interviewer and purpose of the interview  Short presentation of the frame for the interview  Presentation of informant:  Role in relation to ACQUIRE-ICD | What role do you have/have you had in relation to ACQUIRE-ICD?  - How did you get this role? | Ask, if you don’t understand the question  Approx. 30 min, audiorecorded  *CFIR: Proces* |
| **Before onset of the project** |  |  |
|  | Try to remember back when you first heard about the project - what did you think about the idea of treating and communicating with patients online? | *CFIR: Characteristics of Individuals* |
|  | What were your expectations to how the patients would respond to the project? | *CFIR: Outer Setting* |
|  | How were you trained for the task?   - *Did you feel well prepared for the task?* | *CFIR: Proces* |
| **The execution of the intervention** |  |  |
|  | How did the patients respond to the project?  *(If the nurse has only included patients, this question is skipped)*  How did you experience communicating with the patients online? | *CFIR: Proces* |
|  | Have you had issues in the intervention which have been specifically difficult or challenging?   - *If yes – are there anything that have been supportive?* - *If yes – has the job been getting easier with routine?* | *CFIR: Characteristics of Individuals* |
|  | If you compare with talking face-to-face with the patients, are there something which have been better or easier in ACQUIRE-ICD?   - *And are there something which have been harder?* | *CFIR: Proces* |
|  | How did you experience following the protocol for the project?  *- Did you succeed with it?*   - *Did you sometimes do more than/less than the described tasks? (e.g. goal-setting)* - *Can you give examples of what you did?* | *CFIR: Proces* |
|  | How did you experience the patients’ engagement with the intervention?   - *How were you able to assess if the patients’ benefitted from the intervention?* - *If positive - Can you give examples of how?* | *CFIR: Outer Setting* |
|  | Is there something you would like to change on the LIVA platform?   - *E.g. technicalities or interface?* | *CFIR: Intervention Characteristics* |
| **Support and interest from management and colleagues** |  |  |
|  | What did your colleagues in the ICD team think of ACQUIRE-ICD?   - *Are there - for instance - someone in the team who thinks that the psycho-educative element is not part of the job for an ICD nurse?* | *CFIR: Intervention Characteristics*  *CFIR: Inner Setting* |
|  | How does your manager support the project? | *CFIR: Inner Setting* |
|  | Which possibilities have you had for discussing issues or networking about ACQUIRE-ICD? | *CFIR: Outer Setting* |
|  | Have you had the possibility for interdisciplinary cooperation during the project?  *- for instance, with medical doctors if you were insecure of adjustment of ICD/medication/other?* | *CFIR: Inner Setting* |
| **Perspectives of the future** |  |  |
|  | How do you experience the patients are coping with online treatment?   - *Do you think the patients have been missing anything?* - *Do you experience that there are subgroups of patients with an ICD who are not suitable for online treatment?* | *CFIR: Outer Setting* |
|  | Have you missed anything - as a nurse - in your online communication with the patients?   - *If yes, can you describe it?* | *CFIR: Characteristics of Individuals* |
|  | How do you see the potential for implementing ACQUIRE-ICD in the future?   - *Possible barriers?* - *Do you see possibilities for improvement of ACQUIRE-ICD?* - *How about other kinds of online treatment for patients with an ICD?* | *CFIR: Inner Setting* |
|  | Do you think ACQUIRE-ICD or similar online treatment could be used for other groups of cardiac patients?   - *If yes – describe?* | *CFIR: Outer Setting* |
| **Debriefing/finishing of interview** |  |  |
|  | We are about to finish the interview.  Is there anything you would like to add before we stop?  Can we contact you if we have further questions? | Thank you on behalf of the project team |
