# Supplementary material for: A web-based intervention for patients with an implantable cardioverter defibrillator – A qualitative study of nurses’ experiences (Data from the ACQUIRE-ICD study)
Source: PEC Innov. 2022 Nov 28;2:100110. doi: 10.1016/j.pecinn.2022.100110 (PMC10194258; doi:10.1016/j.pecinn.2022.100110)
Supplement: Supplementary file 2 — Coding process [file mmc2.docx]

Appendix B.

Appendix B. Examples of codes, subcategories, categories, and theme from content analysis.

| Theme | Between traditional nursing and modern eHealth | | | |
| --- | --- | --- | --- | --- |
| Category | Online communication challenges patient contact | | Comprehensive intervention | |
| Subcategory | Personal relation matters | Face-to-face is important | Toolbox of patient information and education is very good | Mixed perceptions of online patient network forum |
| Code | Patients benefit from personal contact | Online communication is a barrier | Materials in toolbox are good | Positive towards patient forum |
| Text | Now, had there been a phone contact with the nurse, I think it would have made a difference. Because I could see that the patient that the data manager called, she thought this was great. | I think sometimes in this study, which is much about psychological dimensions, you can miss out on things because you can´t see the patients or their body language, mimic and so on compared to when you have them face-to face. | About the vodcasts, my perception is that it has been very beneficial for the patients. Because you can watch them when it fits, and you are ready. And it is really nice that you can watch them with your spouse, and they can sit together and talk about it. | I believe it is smart with such a forum for ICD patients where they can write stuff, but I actually don’t know if they have used it. |
